# Whole-genome characterization in pedigreed non-human primates using genotyping-by-sequencing (GBS) and imputation

**DOI:** 10.1186/s12864-016-2966-x

**Published:** 2016-08-24

**Authors:** Benjamin N. Bimber, Michael J. Raboin, John Letaw, Kimberly A. Nevonen, Jennifer E. Spindel, Susan R. McCouch, Rita Cervera-Juanes, Eliot Spindel, Lucia Carbone, Betsy Ferguson, Amanda Vinson

**Affiliations:** 1 Primate Genetics Section, Oregon National Primate Research Center, Beaverton, OR USA; 2 Oregon Health & Science University, Portland, OR USA; 3 Section of Plant Breeding and Genetics, School of Integrative Plant Sciences, Cornell University, Ithaca, NY USA

**Keywords:** Whole-genome sequencing, genotyping-by-sequencing, imputation, macaque, pedigree

## Abstract

**Background:**

Rhesus macaques are widely used in biomedical research, but the application of genomic information in this species to better understand human disease is still in its infancy. Whole-genome sequence (WGS) data in large pedigreed macaque colonies could provide substantial experimental power for genetic discovery, but the collection of WGS data in large cohorts remains a formidable expense. Here, we describe a cost-effective approach that selects the most informative macaques in a pedigree for 30X WGS, followed by low-cost genotyping-by-sequencing (GBS) at 30X on the remaining macaques in order to generate sparse genotype data at high accuracy. Dense variants from the selected macaques with WGS data are then imputed into macaques having only sparse GBS data, resulting in dense genome-wide genotypes throughout the pedigree.

**Results:**

We developed GBS for the macaque genome using a digestion with *PstI*, followed by sequencing of size-selected fragments at 30X coverage. From GBS sequence data collected on all individuals in a 16-member pedigree, we characterized high-confidence genotypes at 22,455 single nucleotide variant (SNV) sites that were suitable for guiding imputation of dense sequence data from WGS. To characterize dense markers for imputation, we performed WGS at 30X coverage on nine of the 16 individuals, yielding 10,193,425 high-confidence SNVs. To validate the use of GBS data for facilitating imputation, we initially focused on chromosome 19 as a test case, using an optimized panel of 833 sparse, evenly-spaced markers from GBS and 5,010 dense markers from WGS. Using the method of “Genotype Imputation Given Inheritance” (GIGI), we evaluated the effects on imputation accuracy of 3 different strategies for selecting individuals for WGS, including 1) using “GIGI-Pick” to select the most informative individuals, 2) using the most recent generation, or 3) using founders only.  We also evaluated the effects on imputation accuracy of using a range of from 1 to 9 WGS individuals for imputation. We found that the GIGI-Pick algorithm for selection of WGS individuals outperformed common heuristic approaches, and that genotype numbers and accuracy improved very little when using >5 WGS individuals for imputation. Informed by our findings, we used 4 macaques with WGS data to impute variants at up to 7,655,491 sites spanning all 20 autosomes in the 12 remaining macaques, based on their GBS genotypes at only 17,158 loci. Using a strict confidence threshold, we imputed an average of 3,680,238 variants per individual at >99 % accuracy, or an average 4,458,883 variants per individual at a more relaxed threshold, yielding >97 % accuracy.

**Conclusions:**

We conclude that an optimal tradeoff between genotype accuracy, number of imputed genotypes, and overall cost exists at the ratio of one individual selected for WGS using the GIGI-Pick algorithm, per 3–5 relatives selected for GBS. This approach makes feasible the collection of accurate, dense genome-wide sequence data in large pedigreed macaque cohorts without the need for more expensive WGS data on all individuals.

**Electronic supplementary material:**

The online version of this article (doi:10.1186/s12864-016-2966-x) contains supplementary material, which is available to authorized users.

## Background

The analysis of whole-genome sequence (WGS) data in non-human primates (NHPs) can play a significant role in advancing the application of genomic medicine to human disease. Potential uses of these data include the identification of novel genetic variants that influence conserved pathways of disease pathology, the development of novel therapeutics that target these variants, and the characterization of variants that influence efficacy and response to therapeutics. Given their high degree of genetic and physiological similarity to humans, and their ubiquity in biomedical research, it is surprising that the use of the rhesus macaque for these purposes has been slow to develop. One likely reason for this delay is the dearth of genome-wide sequence information on sufficient numbers of animals to support such studies, which typically require large numbers of phenotyped and genotyped subjects. However, the collection of dense sequence data in large cohorts remains a formidable expense, and a cost-effective solution to this problem is needed if we are to reap the full benefit of NHP models in both basic and preclinical research.

Whole-genome sequencing of large cohorts remains a very expensive undertaking, both now and likely long after we achieve the $1,000 per genome benchmark. Several sequencing strategies have been developed to address this problem, each of which strikes a different balance between sequencing costs, sequence depth, and coverage across the genome. Although deep WGS is the most unbiased and comprehensive method for surveying genetic variants [1], at approximately $2000/genome for standard 30X coverage, its cost remains prohibitive in the foreseeable future for large cohort studies. A second strategy aims to cover the whole genome but at greatly reduced depth (i.e., “low coverage sequencing”), which lowers costs to $100-400/genome. However, this approach reduces the accuracy of resulting genotype data, particularly for smaller studies of rare or low-frequency variants [2]. A third strategy is to sequence only a portion of the genome, i.e., “reduced representation” approaches, which offers a compromise between sequencing depth and breadth of coverage. The most common of the reduced representation approaches is whole-exome sequencing, currently ~ $300/genome for 30X coverage, in which a commercial hybridization kit is used to capture genomic fragments enriched for exons in protein-coding genes. While this approach produces coverage of genomic regions that are of interest to many Mendelian diseases, coverage of regulatory elements or other non-coding regions is sacrificed. Moreover, most commercial exome capture tools are designed for humans or rodents, and thus will miss some portion of the NHP exome.

More recently, a reduced representation approach called genotyping-by-sequencing (GBS) has lowered the cost per genome dramatically, by taking advantage of classical molecular biology methods that capture a more evenly distributed subset of the genome. In the GBS method, restriction enzymes are used to cleave the genome at sequence-specific cut sites, and the resulting fragments are sequenced to the desired coverage. While these fragments still represent only a small portion of the genome, they can be distributed more evenly than in other methods such as exome capture. Importantly, because the GBS approach does not require proprietary capture technology and can be highly multiplexed, costs can be reduced to as little as $30 per genome, and this approach can be applied to species that lack available commercial arrays. This approach has been applied to many agricultural and other economically important species to construct dense genetic linkage maps and identify QTLs [3–8], to improve genome assemblies [9], and to investigate population structure, diversity, and evolutionary history [10–12].

Further gains in the amount of sequence information obtained at the lowest possible cost could be achieved by combining GBS data with imputation, particularly for NHP cohorts with pedigree information. In this approach, WGS data collected in selected individuals within the pedigree are used to impute dense genotypes into their many relatives, in which only sparse genotype data (e.g., obtained by GBS) has been collected. These sparse data from GBS are used to anchor the imputation of genotypes at intervening and more densely spaced loci across the genome, by leveraging information on expected allele-sharing among relatives. This strategy is appealing for many captive NHP breeding colonies, where deep and well-defined pedigrees could permit extremely cost-effective, whole-genome characterization. However, the selection of the most informative animals in the pedigree for WGS is expected to have a large impact on the success of this approach, and studies addressing optimal selection strategies have only been published for human pedigrees, which are typically much smaller and less complex than those characterized for NHP cohorts.

While WGS combined with GBS and imputation presents a significant opportunity for obtaining dense sequence data at minimal cost, this approach has not yet been applied to a pedigreed NHP cohort. Thus, our objectives were to 1) develop a reliable GBS method in the macaque genome to support pedigree-based imputation; 2) assess the extent and accuracy of dense marker data imputed from WGS using sparse marker data from GBS; and 3) compare the extent and accuracy of imputed dense marker data among different strategies commonly used to select individuals for WGS, and among different ratios of WGS to GBS individuals in the pedigree. Here, we show that a *PstI* digest in the macaque genome produces >20,000 high-quality sparse variants that are suitable for use in imputation. We further show that the pedigree-based “Genotype Imputation Given Inheritance” imputation approach (i.e., “GIGI”; [13]), combined with the GIGI-Pick method [14] of selecting individuals for WGS, allowed us to impute an average 3.7 million variants per individual at >99 % accuracy, or alternatively, an average of 4,458,883 variants at a slightly more relaxed threshold yielding >97 % accuracy, among a total 7,655,491 sites of variation spanning all 20 macaque autosomes, using only four individuals with WGS and 12 individuals with GBS. This strategy represents a reasonable tradeoff between sequencing costs, and the amount and quality of dense sequence data obtained on as many individuals as possible.

## Methods

### Animal care and welfare

All macaque samples used in this study were collected during routine veterinary care procedures approved by the Institutional Animal Care and Use Committee of the Oregon Health & Science University (Protocol Number: IS00002621); these samples were obtained from the Oregon National Primate Research Center (ONPRC) DNA Biobank. Animal care personnel and staff veterinarians of the ONPRC provide routine and emergency health care to all animals in accordance with the Guide for the Care and Use of Laboratory Animals, and the ONPRC is certified by the Association for Assessment and Accreditation of Laboratory Animal Care International.

### Pedigree configuration and validation

**Fig. 1:**
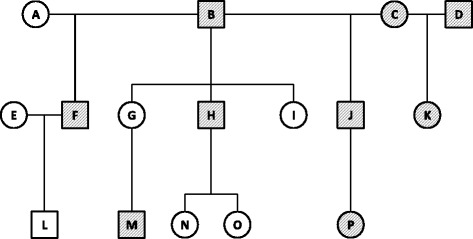
Pedigree diagram of the 16 macaques included in this study. Macaques with whole genome sequence data are shaded; all subjects have GBS data

We selected 16 closely related Indian rhesus macaques from the larger ONPRC colony pedigree as the focus of this study (see Fig. 1). These animals were selected to represent the most common relationships in the colony, including parent/offspring, half-sibling, half-avuncular, half-cousin, and grandparent/grandchild relationships. Because assumed pedigree relationships may prove to be incorrect when comprehensive genotype data are examined, we explored the accuracy of our focal 16-member pedigree using a set of ~5,000 markers on chromosome 19 generated from our GBS sequencing experiments, employing algorithms that assess Mendelian consistent error both pairwise between relatives and within families, as implemented in PedCheck [15] and GIGI-Check [16] software. No significant departures from expected patterns of allele-sharing were noted, confirming the validity of the pedigree configuration depicted in Fig. 1. Initially, nine animals were selected using an ad hoc approach for WGS in this study, based on their position within the pedigree.

### Genomic DNA isolation and quantification

Genomic DNA (gDNA) was extracted from 3 ml of whole blood using the ArchivePure DNA Blood Kit (5 Prime, Hilden, Germany), following the manufacturer’s recommendations. Genomic DNA was quantified with the Qubit High Sensitivity dsDNA Assay (Thermo Fisher Scientific, Waltham, MA).

### Genotyping-by-sequencing (GBS)

To determine the optimal restriction enzymes for conducting GBS in rhesus macaques, we first performed *in silico* mapping of cut sites using the MacaM rhesus macaque reference genome [17], to identify enzymes predicted to produce 60,000-100,000 DNA fragments in the 200–500 bp size range, while also minimizing the presence of repeat sequences (e.g., retrotransposons, DNA satellites). We initially tested the enzymes *ApeKI*, *BglII*, *EcoRI*, *HindIII*, *PspXI*, *PstI,* and *SalI*, ultimately selecting *BglII* and *PstI* as the two enzymes most likely to meet these criteria. We then generated GBS libraries based on these 2 enzymes using a modified version of the method described by Elshire et al. [18]. Specifically, to create the adaptors, oligonucleotides for the top and bottom strands for each barcoded adaptor and for the two common adaptors (one for *BglII* and one for *PstI)* were paired and annealed in 1X Annealing Buffer (20 mM NaCl, 10 mM Tris–HCl pH 7.5, 2 mM MgCl_2_) using a thermal cycler (3 min at 95 °C, ramping down 1.6C/min for 44 cycles, cool to 4 °C). All adaptors were quantified with the Qubit Broad Range dsDNA Assay (Thermo Fisher Scientific, Waltham, MA.). Each of the 32 barcoded adaptors was then paired with a common adaptor at a 1:1 ratio. Each of the 16 genomic DNAs was digested with *BglII* and *PstI* in separate reactions. All 32 reactions (500 ng DNA, 10 U enzyme, in 20 uL volume) were incubated for 2 h at 37 °C, and digests were ligated (400 U T4 DNA Ligase (New England Biolabs, Ipswich, MA) to adaptor mixes (4.5 ng *BglII*, 15 ng *PstI*, in 50 μl volume) for 1 h at 22 °C. Four (4) μl from each ligation reaction was combined into two separate pools, one per enzyme. Both pools were cleaned with DNA Clean and Concentrator (Zymo Research, Irvine, CA) and eluted in 50 μL. Following amplification parameters in Elshire et al. [18], PCR was performed on 10 μl of each pool (Q5 High Fidelity 2X MM (New England Biolabs, Inc.), 25 pmol of each primer, in 50 μl volume) using Primers A and B, to extend and complete the sequencing adaptors. Libraries were purified using the Qiaquick PCR Purification Kit (Qiagen,Valencia, CA.), quantified with the Qubit High Sensitivity dsDNA Assay (Thermo Fisher Scientific, Waltham, MA) and evaluated with the Bioanalyzer High Sensitivity Assay (Agilent, Inc.). A one-sided 0.8X size selection with AMPure XP beads (Beckman-Coulter, Brea, CA) was used to enrich larger size fragments. Libraries were sequenced on an Illumina NextSeq500 at the Oregon Health & Science University Massively Parallel Sequencing Shared Resource to 30X coverage with paired-end 150 bp reads.

### Whole-genome sequencing

Per sample, 1 μg of gDNA was sheared using a Bioruptor UCD200 (Diagenode, Denville, NJ), generating fragments around 300 bp. Libraries were constructed using the NEXTflex DNA Sequencing Kit and NEXTflex DNA barcodes (BIOO Scientific, Austin,TX) following the manufacturer’s instructions. Briefly, the ends of the sheared gDNA were repaired and adenylated, then ligated to barcoded adaptors using the reagents provided. Next, fragments of 200–400 bp were excised from a 1 % agarose gel. The products were amplified by PCR using 8 cycles, then purified using 1X AMPure XP beads (Beckman-Coulter, Brea, CA.). The final libraries were quantified with the Qubit High Sensitivity dsDNA Assay (Thermo Fisher Scientific, Waltham, MA) and evaluated using the 2100 Bioanalyzer High Sensitivity Assay (Agilent Technologies, Santa Clara, CA). Libraries were sequenced on a HiSeq3000 at the Oregon State University Center for Genome Research and Biocomputing to produce 30X coverage with paired-end, 150 bp reads.

### Analysis of sequence data

Both whole-genome and GBS data were processed using the best practice recommendations from the Broad Institute’s Genome Analysis Toolkit (GATK; [19, 20]), adapted for rhesus macaque. Briefly, paired-end reads were trimmed using Trimmomatic [21], and aligned to the MacaM rhesus macaque reference genome [17], using Burrows-Wheeler Aligner [22]. BAM post-processing included local re-alignment around indels using GATK [19, 20]. GATK’s HaplotypeCaller was used to produce VCF files, followed by genotype calling using GenotypeGVCFs. For the latter, a score of 20 was used as the confidence threshold for calling and emitting variants. The resulting VCF was filtered at the site level using the following criteria: quality by depth (QD < 5.0), strand bias (FS > 15.0), mapping quality (MQ < 50.0), proximity to the read end (ReadPosRankSum < −8.0), the difference in mapping quality between reference and alternate reads (MQRankSum < −12.5), and single nucleotide variant (SNV) clusters of three SNVs within a 10 bp span. In addition, SNVs located within repetitive regions, identified using RepeatMasker (http://www.repeatmasker.org), were removed. In addition to these filters, any individual genotypes were removed if either the depth was less than 10, or genotype quality less than 20. The analyses also employed Picard tools [23] and FASTQC [24] for quality control of the raw data, JBrowse [25] to visualize data, and BEDTools [26] to evaluate SNV and imputation marker distribution. Sequence data were managed and analyzed using DISCVR-Seq [27], a LabKey server-based system [28].

### Imputation strategy

We focused on chromosome 19 as a test case in order to develop an analytical pipeline that could be applied to all the remaining chromosomes. We performed imputation using the method of GIGI (“Genotype Imputation Given Inheritance”; [13]), as this method has been successfully used to impute genotypes with high accuracy in extended human pedigrees. This approach infers inheritance vectors (IVs, representing shared chromosomal segments) at sparse marker locations conditioned on observed sparse marker genotypes, and then infers IVs at dense marker locations conditioned on the sparse marker IVs, together with the genetic map. A posterior probability distribution is then estimated for each missing genotype at a dense marker position, conditioned on observed genotypes at all dense marker positions, corresponding allele frequencies, and IVs corresponding to dense markers. In the last step, genotypes may be called using these estimated probabilities, based on user-defined thresholds. We estimated inheritance vectors according to the algorithm of [29], as implemented using a Markov-Chain Monte-Carlo (MCMC) sampler in the gl_auto function in the software package for genetic epidemiology MORGAN 3; available at [30]. The GIGI approach has been implemented in a software package of the same name, and is available at [31].

To characterize the sparse set of markers needed to guide the imputation of dense marker genotypes on chromosome 19, we identified a set of markers that could be detected reliably by GBS among most macaques. Accordingly, we selected a set of high-quality SNVs that, 1) were sequenced to at least 20X depth across the majority of GBS libraries and for which genotypes could be called in at least 50 % of individuals, 2) were spaced evenly across the genome, 3) had minor allele frequencies (MAF) >0.25, and 4) were in excess of what was needed to meet the desired goal of ~0.5–1.0 cM average marker spacing. We refer to these as “framework” markers, as discussed in Cheung et al., 2013 [13]. Using this approach, the desired spacing can be maintained in an approximate fashion, even when individuals are missing a substantial amount of genotype data, an outcome characteristic of the GBS method [32]. Second, to limit the computational time required, we selected a second set of SNVs from our WGS data that were evenly spaced but more densely distributed than the framework markers. We designated these as our “dense” markers, and we attempted to impute these into animals having only sparse framework marker genotypes from GBS. These dense markers were selected from the set of all high-confidence SNVs identified in our cohort.

To determine the success of imputing dense marker data into animals having only sparse framework marker data, we evaluated the accuracy of imputed alleles, defined here as the proportion of alleles imputed correctly among all attempted allele calls at that position, such that a correctly imputed allele is concordant with the allele call from either WGS or GBS sequence data (i.e., “genotype concordance”). We additionally define rare variants as those having only 1 copy among a total of 30 chromosomes (i.e., singletons, present at ~3 % frequency in this dataset) that we have sequenced to date, including the 9 individuals discussed in this paper and an additional 6 unrelated Indian rhesus macaques (unpublished data). We define accuracy of imputation for rare variants as the proportion of rare alleles imputed correctly among all rare variant heterozygotes called from either WGS or GBS sequence data.

To evaluate differences in imputation success associated with increasing the number of individuals with WGS in the pedigree, we assessed accuracy of imputed variants when using from 1–9 animals with WGS data to impute variants into the remaining pedigree members with only sparse GBS data. Individuals with WGS data were added consecutively in the following order: B, H, J, F, M, K, P, C, D (see Fig. 1). Thus, the first scenario used only the most informative animal (B) with WGS to impute genotypes into the remaining 15 animals within the pedigree. Subsequent scenarios retained the previous animal(s), added the next most informative animal, and imputed genotypes into the remaining animals within the pedigree. This procedure was conducted iteratively, until all 9 animals with WGS were used to impute genotypes into the remaining 7 animals in the pedigree. We used the GIGI-Pick algorithm [14] to rank our 9 animals with WGS. This algorithm calculates a metric of coverage, defined as the expected percentage of allele copies called for a variant at a random locus, conditional on fixed IVs for a specific choice of individual(s), and then iteratively selects those individuals with the highest coverage, calculated by integrating over all possible genotype configurations within a given pedigree. We ran the algorithm in genome-wide mode, which requires only the pedigree structure to prioritize individuals for WGS. This algorithm is implemented in the suite of software based on the GIGI approach, and is available at [33]. We evaluated accuracy of imputed genotypes for each of our recipient macaques by masking all non-framework genotypes, and comparing imputed genotypes to masked genotypes obtained from either WGS or GBS data, depending on the data available for each recipient. Specifically, imputed genotypes were compared to genotypes from WGS where available, but for recipients with only GBS data available, imputed genotypes were compared to genotypes at any SNVs that were not designated as framework markers. Imputed genotypes were called using allele frequencies established from all Indian-origin rhesus macaques sequenced to date at the ONPRC as a reference, excluding related individuals (*n* = 12).

To evaluate differences in imputation success associated with different sequencing strategies, we compared the accuracy of genotypes imputed by GIGI on chromosome 19, among 3 different selection strategies, including GIGI-Pick and two common heuristic methods. These 2 methods include whole-genome sequencing of 1) pedigree founders only, or 2) the most recent generation (i.e., individuals typically located at the bottom of the pedigree). To compare the different strategies, we examined accuracy for the scenario in which dense markers from 3 animals selected for WGS are imputed into the remaining 13 pedigree members with GBS data, based on using individuals B, H, and J (GIGI-Pick selections), B, C, and D (“Founders”), and M, P, and K (“Pedigree bottom”) strategies (see Fig. 1). Genotypes were imputed using the GIGI algorithm, based on allele frequency information inferred from the animal set described above. Imputed genotypes were called by 2 methods: by accepting only those above a user-defined probability threshold (“threshold” method) or as the most probable genotype at that position (“most likely” method). For the “threshold” method, we required a 0.99 probability to call both alleles at a given site, and a 0.98 probability to call one of two possible alleles.

To evaluate the impact of missing data on the density of the framework marker panel and downstream effects on the accuracy of imputed genotypes, we evaluated imputation accuracy at 4 different framework marker panels on chromosome 19. We first limited sites to those with MAF >0.25 that were replicated in both GBS and WGS data, since robust variants with high MAF should provide more informative framework markers for imputation. We then reduced these further to include only sites where either 4 of 16 subjects had passing genotypes (1027 markers), 8 of 16 subjects had passing genotypes (811 markers), or 16 of 16 subjects had passing genotypes (325 markers). Finally we created a set of 2,737 markers designed to include every variable position covered by the GBS data, with a genotype called in at least one subject, in order to maximize the possible number of framework markers.

## Results

### Whole-genome sequencing and variant calling

We obtained an average 566,035,688 read pairs per sample (range 495,617,772 − 735,313,000) for each of the 9 individuals with WGS data. These reads were aligned to MacaM [17], to produce an average 27X coverage across the genome (range 24-33X). From these reads, a total of 10,193,425 high-confidence SNVs were identified across all 9 individuals, with an average of 5,037,341 variants detected per individual. The transition/transversion ratio (Ti/Tv) observed in this study was 2.17, consistent with observations in larger macaque cohorts (unpublished data). This set of sites served as the source of our optimal dense marker set, as described in Methods.

### Genotyping-by-sequencing and variant calling

**Fig. 2:**
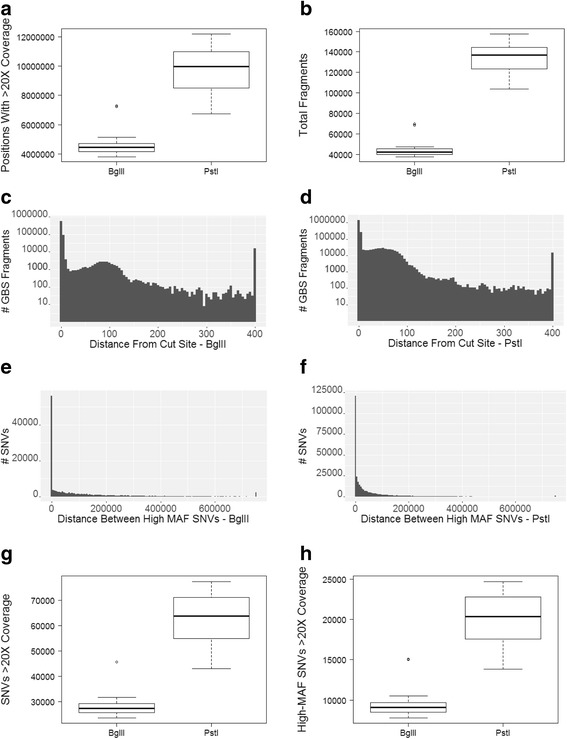
Evaluation of GBS library coverage and SNVs. **a** The number of positions with ≥20X coverage; **b** Total contiguous fragments with >20X coverage; **c** Distance between each GBS fragment and nearest predicted cut site for the *BglII* libraries (all fragments > 400 bp are grouped into a single bin); **d** Distance between each GBS fragment and the nearest predicted cut site for *PstI* libraries; **e** Distance between high MAF (> 0.25) SNVs in *BglII*; **f** Distance between high MAF SNVs in *PstI*; **g **Total SNVs detected per enzyme, and **h** total SNVs with MAF > 0.25

For each of the 16 pedigree members, we prepared and sequenced GBS libraries based on individual digests for both *BglII* and *PstI*. Among *BglII* libraries, we obtained an average 3,754,352 reads per sample, resulting in an average 4,734,354 base-pairs (bp) from 45,597 fragments with at least 20X coverage per sample (equivalent to 0.17 % of the genome). In contrast, among *PstI* libraries, we obtained an average 5,686,709 reads per sample, resulting in an average 9,772,130 bp from 134,314 fragments with ≥20X coverage per sample (equivalent to 0.35 % of the genome) (Fig. 2a-b). Notably, although the *PstI* libraries originally had ~1.5X more reads than *BglII* libraries, they had ~3-fold the number of fragments with high-depth coverage. While the vast majority of GBS fragments were adjacent to the predicted restriction enzyme cleavage site, a small number appeared to be distant from these sites (Fig. 2c-d). While these results may reflect off-target sequencing, it is also possible that they reflect genomic variation altering restriction sites in one or more individuals.

We next determined the number of high-quality SNVs available from each digest that would be suitable for use as framework markers in imputation. We first restricted SNVs to those with >20X coverage, leaving a total of 29,449 high-confidence variants in the *BglII* samples, and 62,619 variants in *PstI* samples. We further restricted these SNVs to only those that were concordant between WGS and GBS data, which included 98.0 % of SNVs for *BglII* (range 97.7–98.4 % among individuals) and 98.7 % of SNVs for *PstI* (range 98.5–98.9 % among individuals). To maximize the probability of the variant being present in as many animals as possible, we also restricted SNVs to only those with MAF >0.25, which further reduced these numbers to an average of 7,399 variants per sample for *BglII* (among 10,775 sites across all samples) and 22,455 variants per sample for *PstI* (among 37,505 sites across all samples), with an average distance between SNVs of 259,690 bp and 78,785 bp, respectively (see Fig. 2e-f). We were not able to call genotypes in all individuals for all SNV sites, due to variation among individuals in sequence quality at each site. From the *PstI* data, all individuals had sufficient data to call genotypes at an average 15,516 (~69 % of 22,455) of these SNVs, but only an average of 4,418 (~60 % of 7,399) of these sites could be called for all individuals from the *BglII* data. Based on the significantly greater numbers of high-quality SNVs across the macaque genome available from *PstI* sequence data, we chose this enzyme for all final imputation analyses.

### Imputation accuracy on chromosome 19 across 3 different strategies for selecting WGS individuals

Our initial tests of imputation focused on chromosome 19 as a small chromosome “test case” in which to validate a large-scale genome-wide approach. To characterize the set of framework markers needed to facilitate imputation on this chromosome, we selected 833 variants spaced ~65 kb apart, from the set of high-MAF sites on this chromosome, characterized as described above. To characterize the set of dense markers to be imputed on this chromosome, we selected 5,010 variants, spaced ~10 kb apart, from the total set of 332,260 sites discovered from WGS data on this chromosome. This reduced and optimized set of dense markers on chromosome 19 was used in all imputation analyses on this chromosome.

**Fig. 3:**
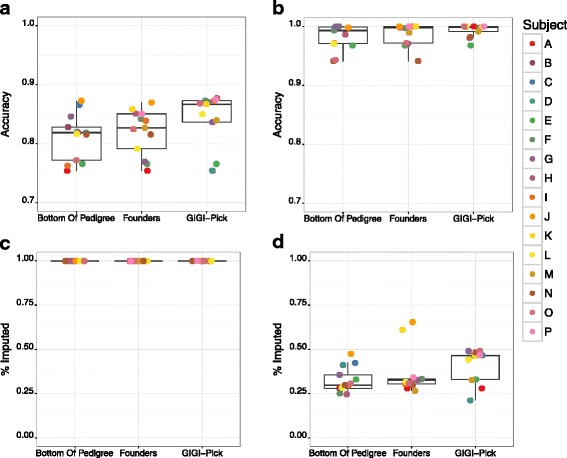
Imputation accuracy on chromosome 19, among different strategies for selecting 3 individuals for WGS. Comparison of imputation accuracy among 3 different strategies for selecting 3 individuals for WGS within the 16-member pedigree: “Bottom of Pedigree” (subjects M, P, K), “Founders” (subjects B, C, D), and “GIGI-Pick” (B, H, J). Imputation of an optimal set of dense markers was conducted for chromosome 19 from the 3 individuals with WGS, into the 13 recipient individuals with GBS data, using the GIGI imputation algorithm with the “Most-Likely” (A) and “Threshold” (B) genotype calling methods. The corresponding fraction of markers imputed are shown for “Most-Likely” (C) and “Threshold” (D) methods. Circles denote individual animals as indicated in the legend; we note that not every individual may be distinguished in these graphs due to overlapping values

Using these chromosome-specific framework and dense marker sets, we evaluated the “Bottom of Pedigree”, the “Founders”, and the “GIGI-Pick” strategies for selecting the 3 most informative of the 9 individuals with WGS data, followed by imputation of dense markers into the remaining 13 individuals, based on their framework marker data from GBS. We imputed genotypes using both genotype-calling methods available in the GIGI software, i.e., the “most likely” genotype for each site (Fig. 3A, C), or assigning only those full or partial genotypes that exceeded a hard probability “threshold” of 0.98 for calling both alleles, or 0.99 for calling a single allele (Fig. 3B, D). The GIGI-Pick selection strategy produced higher median accuracy of imputed genotypes than either of the other strategies, at 86.7 % (“most likely” method, ML) or 99.9 % accuracy (“threshold” method, THR), compared to median accuracy of 81.9 % (ML) or 99.3 % (THR) in the “Bottom of Pedigree” strategy, and 81.9 % (ML) or 99.8 % (THR) in the “Founders” strategy. By design, the “most likely” method will always call a genotype at all markers attempted (Fig. 3C). In contrast, while the “threshold” genotype calling method resulted in considerably higher accuracy across all selection strategies than the “most likely” method, the number of genotypes called was also reduced considerably, with the proportion of markers imputed ranging from a median 29.8 % in “Bottom of Pedigree”, to 32.7 % in “Founders”, and 46.4 % in GIGI-Pick. Within the GIGI-Pick strategy, individuals A, D, and E display lower accuracy, and a lower proportion of imputed variants, likely due to being unrelated to all individuals with WGS in this analysis, i.e., B, H, and J. We note that an unavoidable limitation of our analyses is the larger number of dense variants used to estimate accuracy between individuals with both WGS and GBS data, compared to those with only GBS data. This limitation may explain the slightly lower accuracy of individuals G and N, in whom small differences were magnified relative to individuals having WGS data for comparison.

### Imputation accuracy on chromosome 19 with increasing numbers of WGS individuals

**Fig. 4:**
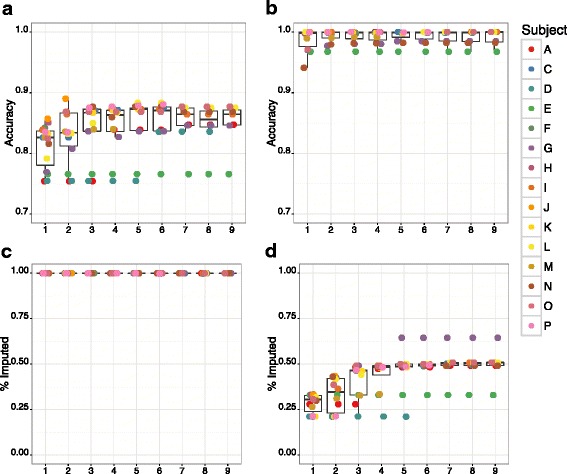
Imputation accuracy on chromosome 19 by total number of individuals selected for WGS. Accuracy for an optimal set of dense markers on chromosome 19, using 1–9 individuals with WGS data, imputed into all remaining pedigree members with GBS data, using the “Most Likely” genotype calling method (A, C) or by assigning genotypes based on hard probability thresholds (“Threshold”) (B, D). Circles denote individual animals as indicated in the legend; we note that not every individual may be distinguished in these graphs due to overlapping values. Individuals with WGS data were ranked by the GIGI-Pick algorithm [14] and used for imputation in the following order: B, H, J, F, M, K, P, C, D (see Fig. 1)

Using the same chromosome 19 framework and dense marker sets, we used the GIGI algorithm to impute our dense markers from 1 to 9 WGS individuals into the remaining 7–15 pedigree members with GBS data, in the consecutive order B, H, J, F, M, K, P, C, and D as ranked by the GIGI-Pick algorithm. We called genotypes using both the “most likely” method (Fig. 4A, C), and the hard probability “threshold” method (Fig. 4B, D). Under the “most likely” method, median accuracy increased from 82.6 to 86.6 % as 1–3 individuals with WGS were added; however, these increases plateaued quickly with only minor gains after 3 WGS individuals were used (Fig. 4A). Maximum median accuracy of 87.2 % was achieved at 5 WGS individuals. We note that even as more WGS individuals were included, two individuals consistently had lower accuracy than other individuals more closely related to the rest of the pedigree. Under the “most likely” method, individual D displayed 76 % accuracy across 1–5 WGS individuals, which increased to 84 % at 6–8 WGS individuals, due to the inclusion of WGS data from K, the child of D. Individual E had 77 % accuracy across all WGS scenarios. As expected, using the “threshold” method, accuracy was considerably higher, with median accuracy of 99.9 % in all scenarios. Under this method, while there were relatively minor differences in accuracy among all scenarios, the median fraction of markers imputed increased significantly from 30.5 to 46.3 % as the first 3 WGS individuals were included, reaching a plateau at 50.8 % when 4–5 WGS individuals were used. Again, founders D and E had the fewest variants called, with only 32.9 % and 21.2 % of positions called, respectively. Based on the optimal tradeoff between accuracy and number of genotypes imputed that was suggested by these results, we selected a ratio of 4 individuals with WGS per 12 individuals with GBS using the”threshold” method for all subsequent analyses.

### Imputation accuracy with varying density of framework markers

**Fig. 5:**
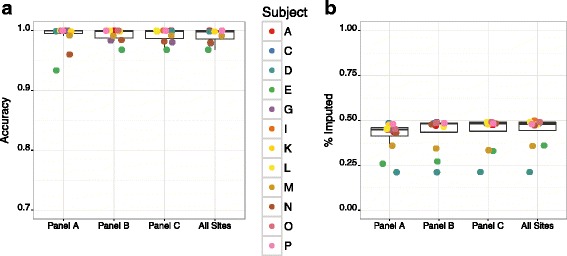
Imputation accuracy on chromosome 19 by density of framework marker panel. A) Accuracy for an optimal set of dense markers on chromosome 19, using 4 individuals with WGS data, imputed into all remaining pedigree members with GBS data. Circles denote individual animals as indicated in the legend; we note that not every individual may be distinguished in these graphs due to overlapping values. Framework marker panels were designed by selecting positions with GBS data that intersect with SNVs of MAF >0.25. Three framework panels were tested: 325 SNVs present in all 16 individuals (Panel A); 811 SNVs present in 8/16 individuals (Panel B); 1,027 SNVs present in 4/16 individuals (Panel C), and 2,737 SNVs that include all sites with high-confidence genotypes in the GBS data (All sites). B) The fraction of dense markers imputed using hard probability thresholds for calling genotypes, as described in Results

As the number of framework markers increased from 325 up to the maximum available of 2,737, there was little variability in median accuracy of imputed genotypes, or in the fraction of markers imputed. Based on the “threshold” genotype calling method, median accuracy was 99.9 % for all marker panels, and the median fraction of markers imputed was 44.7 % for Panel A, 48.0 % for Panel B, 48.2 % for Panel C and 48.0 % for All Sites (Fig. 5A-B). Only individuals E and N showed initial improvement in accuracy with increasing marker density, but these improvements plateaued quickly. Individuals E, N, and G consistently exhibited lower accuracy overall than all other individuals across all marker density panels (again likely due to differences in the number of markers used to compare accuracy between WGS and GBS individuals), while individuals D, E, and M were consistently more poorly imputed than all others. For individuals D and E, this is likely due to being unrelated to any of the 4 WGS individuals used for imputation, i.e., B, H, J, and F. Importantly, both accuracy and the fraction of markers imputed were relatively consistent across a range of framework marker sets, and robust to a wide range of missing genotype data. Gains in accuracy and fraction of markers imputed were negligible when using all GBS data available.

### Imputation rate and accuracy across allele frequencies on chromosome 19

Using the threshold genotype calling method, among a total 96 instances on this chromosome, private/rare alleles were imputed a total of 38 times (~40 %), with 100 % accuracy, but were not called 58 times (~60 %; see Additional file 1: Figure S1). Across all other allele frequencies, only slightly lower accuracies were obtained (>99.7 %), while imputation rates increased with allele frequency, as expected. Alleles at frequencies of 0.00 − 0.25 were imputed 20.7 % of the time; at 0.25 − 0.50 frequencies, 30.3 % of the time; at 0.50 − 0.75 frequencies, 30.3 % of the time; and alleles with frequencies >0.75 were imputed 47.2 % of the time (see Additional file 1: Figure S1).

### Imputation of dense markers across the genome

**Fig. 6:**
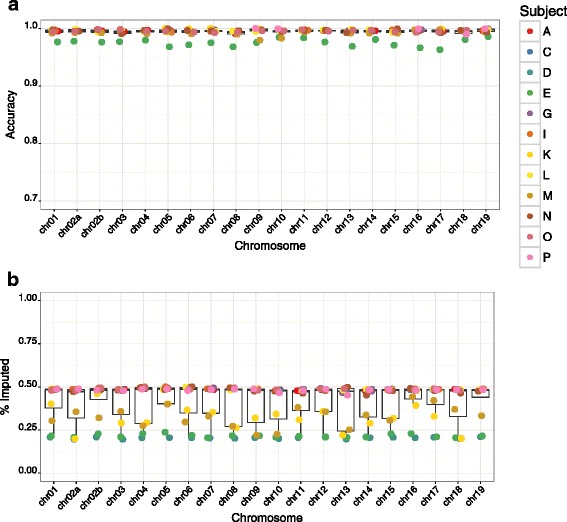
Imputation accuracy across the genome at a comprehensive set of dense markers. A) Accuracy of alleles imputed across all autosomes at 7,655,491 possible sites, and B) fraction of markers which were imputed using hard probability thresholds for calling genotypes, as described in Results. Data represent accuracy of alleles at dense markers for 12 pedigree members with GBS data, imputed from individuals B, H, J, and F, called using the “Threshold” method for calling genotypes. Circles denote individual animals as indicated in the legend; we note that not every individual may be distinguished in these graphs due to overlapping values

To evaluate our imputation strategy across the whole genome, we employed the same criteria outlined above to generate framework marker sets for each of the 20 macaque autosomes. The number of framework markers per chromosome ranged from 435 to 1,450, totaling 17,158 across the genome, with mean spacing between framework markers among all chromosomes of ~159 kb (81 kb-253 kb). At this stage, we modified our dense marker set to a total possible 7,655,491 sites across the macaque genome, by including variants discovered previously from WGS data in an additional 6 unrelated ONPRC Indian rhesus macaques (in preparation), and by removing any variants with insufficient allele frequency information to allow imputation. Based on the strategy identified by our analyses as optimal, we used the first 4 individuals among our 9 with WGS as ranked by the GIGI-Pick algorithm, and imputed variants at this comprehensive set of dense markers into the remaining 12 pedigree members, based on the “threshold” calling method (Fig. 6). Per chromosome, median accuracy ranged from 99.4 to 99.8 % and the median fraction imputed ranged from 47.1 to 48.8 % with an average of 3,680,237 correctly imputed variants per subject. However, several individuals had significantly lower overall numbers of variants imputed at this level of accuracy, including D, E, K, and M (range 1,563,908-2,524,889), and individual E also consistently exhibited the lowest accuracy across all chromosomes at 96.3-98.5 %. These data were imputed using a probability threshold of 0.98 or 0.99 to call both or only one allele in a genotype, respectively; at a more relaxed threshold of 95 % confidence required to call both alleles, and 98 % confidence to call a single allele, we obtained an average 4,458,883 (~58 %) correctly imputed variants with an overall accuracy of > 97 %.

### Genome-wide imputation rate and accuracy across allele frequencies

Using the threshold calling method, 17.3 % of 27,761 private/rare alleles were imputed with 99.9 % accuracy. Across all other allele frequencies, only slightly lower accuracies were obtained (> 99.7–99.9 %), while imputation rates increased with allele frequency, as expected. Alleles at frequencies of 0.00 − 0.24 were imputed 15.5 % of the time; at 0.25 − 0.49 frequencies, 23.1 % of the time; at 0.50 − 0.74 frequencies, 27.3 % of the time; and alleles with frequencies ≥0.75 were imputed 44.3 % of the time.

### Post hoc comparisons of this approach to low-coverage WGS sequencing

After completing the analyses described above, we compared the genotype accuracy and density described in our WGS/GBS approach, to other low-coverage sequencing approaches, with or without imputation, but limited to a similar total sequencing cost. As of this writing, low-coverage sequencing remains more expensive than GBS per individual, so alternative, equivalent-cost approaches that substitute low-coverage sequencing would need to compensate by reducing or eliminating higher-coverage sequencing elsewhere in the study design. For example, one individual with 16X coverage may be used to impute into four individuals with 4X data. To simulate this 16X/4X imputation situation, we down-sampled the 30X WGS data from our analysis to 16X or 4X coverage and used these data to call genotypes as described above. We then compared the accuracy and number of genotypes obtained from these down-sampled data to the original 30X data. As shown above, only a few hundred framework markers per chromosome are sufficient for imputation. While the simulated 4X data provided genotypes at more than enough sites per sample to support imputation, we note that overall genotype concordance between 4X and 30X data was comparatively low (96.8 %), due to the lower read depth of the 4X data. We did find very high concordance between genotypes called in the 16X and 30X data (>99 %); however, the total number of genotypes that could be called was significantly greater in the 30X data, with an average of 8,549,800 per sample, compared to only 5,030,830 per sample in the 16X data, i.e., a 70 % increase. This result suggests that including animals with higher-coverage sequencing will allow the imputation of significantly more genotypes, more extensively throughout the pedigree, than a moderate/low coverage imputation approach. We note that another possible imputation approach using one 28X individual to impute variants into 4-1X individuals would cost the same as the 16X/4X approach (i.e., both total to 32X coverage), but would require high-confidence information *a priori* on colony or population allele frequencies in order to call variants with sufficient accuracy from 1X data to produce framework markers. Similarly, sequencing all 5 animals at 6X without imputation would require the same high-confidence information *a priori*. This information does not currently exist for the rhesus macaque, and many novel model organisms face this same limitation.

## Discussion

The rhesus macaque is widely used in academic biomedical research, primarily due to its utility as a model of human HIV infection and pathology. Although this species is well-known for the susceptibility to HIV that it shares with humans [34, 35], it is not widely appreciated that macaques naturally display variation in susceptibility to a broad spectrum of diseases and disorders that mimic those found in humans, e.g., dyslipidemia, addictive disorders, macular degeneration, and anxiety [36–42]. While the macaque was identified early as a high priority for assembly of a reference genome, and a draft genome was subsequently published in 2007 [43], the systematic application of genome-wide data in the macaque to the study of human health and disease has yet to materialize. Moreover, technologies such as large scale SNP genotyping arrays that are taken for granted in humans and many rodent models, have never been developed for the macaque.

Since 2007, next-generation sequencing technology has speeded the collection of genomic data at steadily decreasing cost, but only a relatively small number of additional macaque genomes have been explored for variation, and none have yet been systematically applied to the study of human disease. This is unfortunate, given that large, outbred pedigreed rhesus macaque colonies at many primate research centers constitute a powerful resource for the genetic analysis of common human diseases. Here, in order to catalyze the application of genomic data in macaques to the study of human disease, we present an approach enabling the relatively affordable collection of accurate, dense genome-wide sequence data in large numbers of pedigreed macaques.

Our approach is based on using a low-cost reduced representation sequencing method (genotyping-by-sequencing, GBS), to facilitate pedigree-based imputation of dense marker genotypes from selected relatives with whole genome sequence data. In this study, we evaluated the ability of 2 candidate restriction enzymes (*BglII* and *PstI*) to produce genomic fragments for GBS, using both *in silico* and empirical methods. When compared to *BglII,* we show that *PstI* captures substantially larger numbers of high-quality variants, including more that are robustly replicated in WGS. Further, we demonstrate that even though *PstI* libraries provide coverage over only 0.35 % of the genome, this coverage produced significantly more variants than are needed to generate the sparse “framework” markers required to support imputation of dense marker data from WGS individuals. Because GBS is able to facilitate the imputation of dense, genome-wide data at very low cost (roughly 15 % the cost of 4X sequencing), it becomes financially feasible to apportion more key individuals in the pedigree to greater depth WGS, resulting in a greater number of high-confidence genotypes throughout the pedigree. Our approach contrasts with alternative approaches that combine high-coverage with low-coverage WGS [44]. However, we note that a “one-size-fits-all” approach may not be possible, due to differences among studies that include, 1) pedigrees of varying size and reliability, 2) variable types and amounts of prior genotype data (i.e., microsatellite or other sparse but informative markers, dense SNP data, exome sequence data, etc.), 3) varying amounts of population-level information (i.e., known vs. unknown population allele frequencies), and 4) potentially different goals (gene-centric coverage, genome-wide coverage for association, genome-wide coverage for linkage analysis, discovery of rare SNVs, or discovery of regulatory, structural, or other variant types, etc.). In addition to these complexities, dollar-for-dollar comparisons are difficult because final costs per individual in terms of genotype numbers and accuracy achieved are typically not reported, and because sequencing costs continue to decline at a rapid rate. Given the minimal amount of prior genome-wide information of any kind in the ONPRC macaque colony, the lack of high-confidence population allele frequency information, and the substantially greater number of variants that can be imputed throughout the pedigree by maximizing the sequencing coverage on many more key individuals, we believe our approach provides optimal genotype information at minimum cost. This approach could be applied to other managed or natural colonies of Indian-origin rhesus macaques with pedigree information but limited or no other data, and potentially to similar groups of other macaque subspecies.

Ultimately, we demonstrate that GBS data can be used to impute genotypes at an average ~3.7 million SNV sites over all 20 autosomes, at >99 % accuracy throughout a 16-member pedigree. This high accuracy was obtained by only calling imputed genotypes above a very strict probability threshold, and therefore comes at the expense of the total number of genotypes imputed. This threshold could be relaxed in applications where a lower accuracy may be tolerated in order to increase the density of genotype information; for example, ~4.5 million variants can be imputed in the same cohort at > 97 % accuracy. However, even at ~3.7 million sites, this number of variants already exceeds the capacity of many state-of-the-art human genotyping chips used for discovery in complex disease as of this writing, including the Illumina Infinium® Multi-Ethnic EUR/EAS/SAS-8 (~1.65 M markers), the Infinium® Multi-Ethnic Global-8 (~1.95 M markers), and the Infinium® Omni2.5-8 Beadchip (~2.7 M markers).

In addition to common variants, we note that we are able to impute rare or private variants at exceedingly high accuracy. Moreover, these results were obtained using very preliminary estimates of population allele frequencies; as more animals are sequenced, this information will improve overall accuracy of allele frequency estimates and therefore the number of genotypes able to be imputed at a specified confidence level, including rare variants. Our goal is to characterize an unbiased, dense set of genome-wide markers throughout the much larger colony pedigree, and for these data to provide flexibility for use in genome-wide association and/or genome-wide linkage approaches, and in candidate gene studies. The amount and quality of genome-wide genotype information obtained using the approach outlined in this paper, combined with the extensive pedigree information available in this macaque colony, will offer powerful support for downstream analysis of both rare and common variant effects on complex traits.

The selection of individuals for WGS that will maximize the accuracy of imputed genotypes throughout the pedigree is a critical component of this approach. We compared GIGI-Pick [14], a pedigree-based statistical approach to prioritizing subjects for WGS, to two other common heuristic methods for selecting individuals for WGS, including sequencing only the most recent generation of the pedigree (“Bottom of Pedigree”), and sequencing only pedigree founders (“Founders”). We show that while high accuracy of imputed genotypes was achieved in all three strategies, on average the GIGI-Pick selection strategy was able to impute a significantly larger number of genotypes than either the “Bottom of Pedigree” or “Founders” approach. It is possible that these 3 selection methods may perform differently for alternative pedigree configurations, e.g., in a more shallow pedigree, sequencing founders or the most recent pedigree members may provide information equivalent to the more formal strategy implemented in GIGI-Pick. However, we note that the GIGI-Pick approach results in a clear advantage even in this small pedigree that extends to only 2 generations, but which includes many of the most common relationships typically found in NHP breeding colonies. Moreover, our results are consistent with those of Cheung et al. [14], in that the GIGI-Pick selection approach substantially outperformed both the “Bottom of Pedigree” and “Founders” (i.e., “PRIMUS” in [14]) approaches in the ability to impute common alleles, although our results indicate more consistent accuracy with the “Founders” approach than with the “Bottom of Pedigree” approach.

Using the WGS individuals ranked in order of priority by the GIGI-Pick algorithm, we also examined the gain in accuracy of imputed genotypes throughout the pedigree achieved by increasing the number of WGS individuals used for imputation. Our results demonstrate that there are excellent compromises available that balance sequencing costs and the ability to obtain dense and accurate marker data. While the number of genotypes that can be confidently imputed increases as more individuals with WGS are included, the most significant gains are achieved using the first 4 WGS individuals in the pedigree, with relatively modest gains thereafter. While this result may not be true for all pedigree configurations, our findings suggest that an optimal tradeoff in this extended pedigree exists at the ratio of 1 individual selected for WGS, per 3–5 relatives selected for GBS, a cost savings of ~66–82 % over 30X WGS of all 6 individuals.

The increase in overall accuracy observed with additional WGS individuals was not shared uniformly among all individuals in the pedigree. We note that D and E remained outliers in the distribution of genotype accuracy throughout virtually all imputation analyses based on the ranking of WGS individuals by the GIGI-Pick algorithm. Individual E is the only founder lacking any relationship to a WGS individual. However, the difficulty of imputation in D may be due to the limited initial selection of WGS individuals located in the far right lineage, i.e., only when K, P, and C are added to J and used for imputation does accuracy rise for D. This result is consistent with the GIGI-Pick approach, which balances the selection of closely related individuals within the pedigree to facilitate phasing of genotypes, with the selection of more distant relatives to increase the chance of observing unique founder alleles [14]. Because of this compromise, we note that when using the GIGI-Pick approach, some pedigree founders may remain unselected when the ability to phase genotypes in one portion of the pedigree produces greater numbers of expected allele calls than does the selection of unique founder alleles from elsewhere in the pedigree. This result also highlights the importance that prior knowledge of phenotypes plays in selecting individuals for WGS. If traits of interest are known to segregate in a particular lineage within the larger pedigree, it may be advisable to manually assign either founders or a close descendant in that lineage for WGS, if neither individual is selected using a more unbiased approach. These results confirm that the success of imputation using this approach is dependent on position in the pedigree, and on the overall ratio of WGS to GBS individuals. As this ratio decreases, entire branches or lineages within larger, more complex pedigrees may receive limited data of lower accuracy. Additionally, with limited information on population allele frequencies (as in this study), pedigree members without any relationship to a WGS individual (i.e., “E”) will be more poorly imputed than those related to a WGS individual. However, as noted above, estimates of population allele frequencies will improve dramatically with the addition of more individuals with either WGS or GBS sequence data, and therefore imputation in individuals such as E should also improve considerably. In general, we expect that with a more realistic pedigree size and better estimates of colony-wide allele frequencies, we will improve our ability to impute a much larger set of variants at >99 % accuracy throughout the pedigree.

The imputation of dense, genome-wide genotypes with high accuracy will allow the unbiased mapping of genetic variants in the macaque genome to disease traits, using either linkage or association approaches. Both of these approaches are important tools in translational research, and should further advance the understanding of human disease already made possible by research in this species. Large pedigreed colonies of macaques, such as the ~4,500 macaques at the ONPRC, provide an almost unequaled resource for translational genetic research, due to their multi-generational pedigree structure and the enriched number of rare and low-frequency variants expected to segregate within this pedigree. Rare and low-frequency variants are expected to play a significant role in human disease [45–48], and we have demonstrated that our approach should discover and impute many of these variants in the macaque genome with high accuracy and at a reasonable cost. Moreover, our findings suggest that this approach can be modified to support specific research goals. For example, it may be beneficial to take advantage of the less accurate but greater number of imputed genotypes provided by a larger set of dense markers during initial discovery of variants either linked to or associated with a disease trait, while fine-mapping or replication of a putative trait locus might employ a reduced, optimal set of dense markers likely to provide greater genotype accuracy over a smaller region of interest.

## Conclusions

We conclude that using a combination of high-coverage WGS and GBS together with pedigree-based imputation, is a feasible and highly cost-effective method for obtaining comprehensive and accurate genome-wide variation throughout a pedigreed cohort.  We demonstrate this approach for the first time in a 16-member extended NHP family, by imputing an average ~3.7–4.5 million variants per individual at 97-99 % accuracy, using only 4 individuals with WGS data and genotypes at only ~17,000 genome-wide markers from GBS data in the remaining 12 relatives.  Future application of this approach in a much larger pedigree will produce a powerful resource for the genetic study of complex disease in NHPs.

## Abbreviations

BWA, burrows-wheeler aligner; CNV, copy number variant; GATK, genome analyzer toolkit; GBS, genotyping-by-sequencing; GIGI, genotype imputation given inheritance; MAF, minor allele frequency; MCMC, Markov Chain Monte Carlo; ML, most likely genotype calling method; ONPRC, Oregon National Primate Research Center; SNV, single-nucleotide variant; THR, threshold genotype calling method; VCF, variant call format; WGS, whole-genome sequencing

## Additional file

Additional file 1: Figure S1. Genotype accuracy based on frequency of alleles imputed. Data represent results of variants imputed at 5,010 markers in 12 pedigree members with GBS data, imputed from individuals B, H, J, and F with WGS data. Genotypes were called using the “Threshold” method for calling genotypes, as described in the main text. (PDF 237 kb)
