# Supplementary figures and images for: Whole-genome characterization in pedigreed non-human primates using genotyping-by-sequencing (GBS) and imputation
Source: BMC Genomics. 2016 Aug 24;17(1):676. doi: 10.1186/s12864-016-2966-x (PMC4997765; doi:10.1186/s12864-016-2966-x)

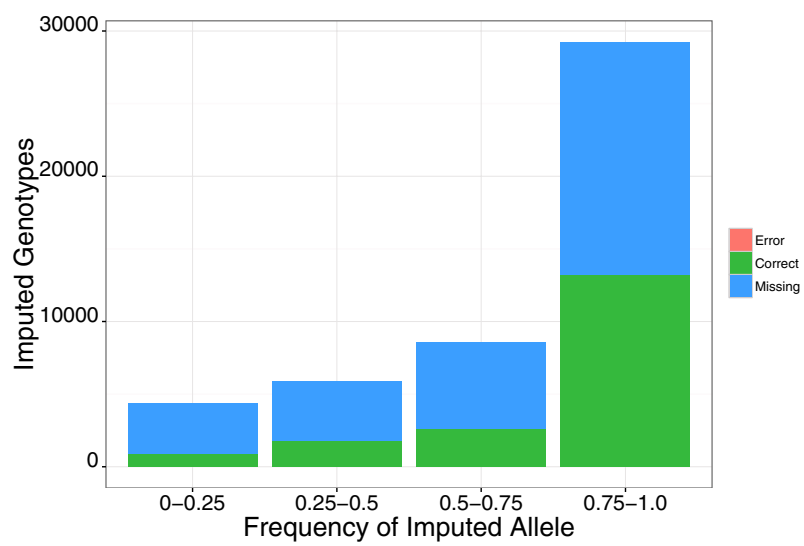

Supplement: Additional file 1: Figure S1. — Genotype accuracy based on frequency of alleles imputed. Data represent results of variants imputed at 5,010 markers in 12 pedigree members with GBS data, imputed from individuals B, H, J, and F with WGS data. Genotypes were called using the “Threshold” method for calling genotypes, as described in the main text. (PDF 237 kb) [file 12864_2016_2966_MOESM1_ESM.pdf]
